# Editorial: Bronchopulmonary dysplasia: latest advances-volume II

**DOI:** 10.3389/fped.2025.1755057

**Published:** 2026-01-05

**Authors:** Shahana Perveen, Jia-Yuh Chen

**Affiliations:** 1 Department of Pediatrics, Northwell Health, New Hyde Park, NY, United States; 2 Division of Neonatology, Cohen Children’s Medical Center, New Hyde Park, NY, United States; 3 Division of Neonatology, Changhua Christian Children’s Hospital, Changhua, Taiwan

**Keywords:** bronchopulmonar dysplasia, complex pathogenesis, morbidity, mortality, preterm infant, survival

Bronchopulmonary dysplasia remains the most common chronic disease of prematurity with high morbidity and mortality (1). With advancement in technology and improving care, extreme preterm infant survival has been on higher side but without improvement in the incidence of BPD which remains stable or even higher with improved survival (2, 3). This underscores BPD's complex, multifactorial etiology and the critical need for continued research into preventive and therapeutic interventions that specifically target its underlying pathophysiology in vulnerable infants (4–6).

The Research topic “*Bronchopulmonary Dysplasia: Latest Advances’ Volume II*”, compiles 13 new and compelling articles. These studies collectively explore the etiology, pathophysiology, preventive strategies, and emerging therapeutic modalities of BPD, all aimed at improving infant survival and long-term outcomes. Multiple studies and meta-analyses demonstrate that Ureaplasma colonization of the respiratory tract or exposure *in utero* is associated with a higher incidence of BPD (7–9). YaBo Mei et al. described the ‘Association of Ureaplasma urealyticum colonization and clinical outcomes in extremely premature infants based on a single center retrospective study and reports that although Ureaplasma urealyticum colonization was associated with longer need for invasive mechanical ventilation but it was not found as an independent risk factor for BPD.

Inflammation has been described in literature as key component in the pathophysiology of BPD (10).

Shams et al. describe the role of TNF-α polymorphism and the susceptibility to BPD in preterm neonate. Current evidence suggests that certain tumor necrosis factor-alpha (TNF-α) gene polymorphisms, are associated with increased susceptibility and severity of bronchopulmonary dysplasia (BPD) in preterm infants (11, 12), but findings are inconsistent across populations and require further validation. This systematic review “*Association of TNF-α Genetic Variants with Neonatal Bronchopulmonary Dysplasia: Consolidated Results*” by Shams et al. based-on total 14 case controlled studied published before Oct 1, 2024. However, this Meta analysis results should be interpreted cautiously as it might be associated with publication bias and heterogeneity of population may affect the link between TNF-α and risk of developing BPD.

Pulmonary hypertension complicates moderate to severe BPD in up to 25%–30% of preterm infants, driven by vascular remodeling, growth arrest, and increased pulmonary vascular resistance (13).

Lindberg described in his Case report “*The rationale of using angiotensin receptor blocker instead of pulmonary vasodilators to treat pulmonary hypertension in bronchopulmonary dysplasia: a case report and literature review*”. This case report highlights the rational of switching pulmonary vasodilator to systemic afterload reduction using losartan, an angiotensin II type 1 receptor blocker with improvement in pulmonary hypertension and crisis. The literature review not only validates an updated understanding of BPD's pathophysiology but also indicates that targeting the renin-angiotensin-aldosterone system, instead of pulmonary vasodilation, could be a new therapeutic approach, but this needs further exploration and research.

Mcomber et al. described the role of “*Predictive Analytics in Bronchopulmonary Dysplasia: Past, Present, and Future*”. This review article relates that leveraging statistical and machine learning techniques, predictive analytics can enhance BPD management by utilizing large clinical datasets to predict individual patient outcomes and by providing personalized interventions for infants at risk of BPD.

Systemic dexamethasone became a traditional therapy in BPD management, widely adopted in the 1990s and early 2000s. Its popularity stemmed from its potent anti-inflammatory effects, its ability to facilitate extubation in ventilator-dependent preterm infants, and compelling evidence from early randomized trials demonstrating a significant reduction in BPD incidence (14). However, concerns regarding neurodevelopmental impairment, particularly the increased risk of cerebral palsy following administration during the first postnatal week, prompted a shift toward lower doses and later initiation (15). Mielgo et al. in their original research article “*Ciclesonide shows a lung-protective effect in neonatal rats exposed to intra-amniotic enterotoxin*” shows that the use of a new glucocorticoid, ciclesonide, can attenuate the alteration of lung structure and pulmonary hypertension in a rat model of chorioamnionitis-induced BPD, with minimal adverse effects on the developing brain. This suggests that ciclesonide could represent a new therapeutic approach for BPD prevention. Nevertheless, long-term data are still needed to confirm its efficacy and safety.

Tang et al. explore the “*Risk Prediction for Bronchopulmonary Dysplasia in Premature Infants under New Diagnostic Criteria*”. This was based on retrospective collected case data from August 2015–2018. This study presented a preliminary risk model for early BPD prediction, which showed good discrimination and calibration in premature infants. However, the limitations of this study's single-center design and limited sample size indicate that future research should be multi-center, incorporating longitudinal follow-up at various time points, to establish a comprehensive and optimal prediction mode.

Early intra-tracheal administration of budesonide with surfactant therapy may reduce BPD incidence and severity, as reported previously (16). However, the findings are not universally consistent (17). “*Meta-analysis of budesonide and surfactant combination for the prevention of bronchopulmonary dysplasia in preterm neonates based on gestational age*” by Ekraminasab et al. describes that intratracheal administration of pulmonary surfactants combined with budesonides can reduce the incidence of BPD, mortality, and PDA. This is a meta-analysis study involving a systematic examination of various online databases.

A randomized trial (DART) protocol advocates for a low-dose dexamethasone with a cumulative dose of 0.89 mg/kg for 10 days (18). “*Enhanced vs. standard low-dose dexamethasone treatment on respiratory outcomes of preterm infants with bronchopulmonary dysplasia*” by Gunes et al. finds that an enhanced low-dose dexamethasone (1.35 mg/kg) may have superior respiratory outcomes compared to the conventional DART protocol without significant increase in short-term adverse effects.

## Conclusion

The collective body of work on “*Bronchopulmonary Dysplasia: Latest Advances*” powerfully illustrates the persistent, multifaceted challenge BPD presents, while simultaneously illuminating a dynamic path forward. These articles showcase remarkable strides, from refining preventative strategies with optimized and more tailored patient care.

In conclusion, the latest advances in bronchopulmonary dysplasia research underscore both significant progress and ongoing challenges in understanding and managing this complex condition. Nonetheless, translating these scientific advancements into routine clinical practice requires rigorous validation, multicenter collaboration, and long-term follow-up. As research continues to unravel the intricate mechanisms underlying BPD, a future where prevention and treatment are tailored to each infant's unique risk profile becomes increasingly attainable, offering hope for healthier lungs and brighter beginnings for our most vulnerable patients.
